# Topological data analysis in single cell biology

**DOI:** 10.3389/fimmu.2025.1615278

**Published:** 2025-09-02

**Authors:** Enrique Hernández-Lemus

**Affiliations:** Computational Genomics Division, National Institute of Genomic Medicine, Mexico City, Mexico

**Keywords:** topological data analysis, single cell biology, persistence homology, simplicial complexes, cell type assignment, systems immunology

## Abstract

Single-cell technologies have revolutionized our ability to interrogate biological systems at unprecedented resolution, revealing complex cellular heterogeneity and dynamic processes that underlie development, disease, and immune responses. However, the high dimensionality and nonlinear structure of single-cell data present substantial analytical challenges. Topological data analysis offers a powerful mathematical framework for capturing the intrinsic shape of data, providing novel insights that complement and extend traditional statistical and machine learning methods. By leveraging tools such as persistent homology and the Mapper algorithm, TDA enables the detection of subtle, multiscale patterns – including rare cell populations, transitional states, and branching trajectories – that are often obscured by conventional approaches. In this review, we explore the theoretical foundations of topological data analysis and examine its emerging applications across single-cell transcriptomics, proteomics, and spatial biology. We highlight how this approach can unveil previously unrecognized biological phenomena, from alternative differentiation paths to complex tissue architectures, and discuss the growing ecosystem of computational tools that support its use. As single-cell datasets become increasingly large and multimodal, topological data analysis stands out as a uniquely robust and interpretable approach, with the potential to deepen our understanding of cellular identity and function in health and disease. TDA is specially suited for fields such as systems immunology since it can capture the complex, nonlinear structures inherent in high-dimensional immune data helping to identify distinct immune cell states, differentiation pathways, and dynamic responses to infection or therapy. This topological perspective complements traditional statistical approaches, providing a robust, scale-invariant framework for uncovering hidden organization within the immune system’s complexity.

## 1 Introduction

Topological data analysis (TDA) has emerged as a powerful mathematical framework for uncovering the intrinsic geometric and topological structure of complex, high-dimensional datasets (1–3). Originally rooted in algebraic topology, TDA provides tools for describing the shape of data, allowing researchers to detect features such as clusters, loops, and voids that traditional statistical or dimensionality reduction methods may overlook (4, 5). In recent years, the application of TDA to biological systems has gained momentum (6), particularly in the field of single-cell biology, where the complexity and heterogeneity of data pose significant analytical challenges.

Single-cell biology aims to dissect biological systems at the level of individual cells, offering insights into cellular heterogeneity, developmental trajectories, and rare cell populations that are obscured in bulk measurements (7–9). Advances in technologies such as single-cell RNA sequencing (scRNA-seq) (10–12), mass cytometry (13–15), and spatial transcriptomics (16–19) have led to the generation of massive, high-dimensional datasets that capture the nuanced variation among thousands to millions of cells. Traditional approaches for analyzing these datasets, including clustering, principal component analysis (PCA), and t-distributed stochastic neighbor embedding (t-SNE), while useful, often impose linear or locally constrained assumptions that can distort the underlying biological structure (20–22).

In contrast, TDA methods are model-independent and inherently multiscale, making them particularly suited to capturing the global organization and hidden structures within single-cell data (23–26). One of the most widely used tools in this space is persistent homology, which quantifies the persistence of topological features across multiple scales, providing a robust summary of the data’s shape (27–30). Another influential technique is the Mapper algorithm, which constructs simplified representations of high-dimensional data by identifying and linking regions of similar local geometry. These methods can illuminate continuous and branching processes, such as cellular differentiation and lineage trajectories, in ways that conventional tools cannot (26, 31–33).

The application of TDA to single-cell biology has led to novel biological discoveries and has complemented existing computational approaches by providing alternative perspectives on the structure of the data. For example, TDA has been used to identify rare or transitional cell states (25, 34–36), to reconstruct developmental processes (23, 37–39), and to map immune responses with high resolution (34, 40). Furthermore, TDA-based visualizations and summaries often serve as intuitive and interpretable models, enabling biologists to engage directly with complex datasets.

Despite these advantages, the adoption of TDA in the broader single-cell community remains limited, in part due to the mathematical complexity of the methods and the relative scarcity of user-friendly software implementations. However, ongoing interdisciplinary collaborations between mathematicians, computer scientists, and biologists are rapidly improving the accessibility and applicability of TDA tools. Efforts to integrate TDA with machine learning and graph-based methods are also expanding the analytical repertoire available for single-cell data (6).

This review aims to provide a comprehensive overview of the state-of-the-art in TDA methods as applied to single-cell biology. We begin by introducing the mathematical foundations of TDA, focusing on concepts such as simplicial complexes, persistent homology, and topological signatures. We then survey key applications of TDA in single-cell transcriptomics, proteomics, and spatial omics, highlighting case studies that demonstrate its utility in revealing biological insights. Attention is also given to software tools and computational frameworks that facilitate the use of TDA in practice.

In addition to reviewing current applications, we discuss the limitations and challenges associated with TDA in the single-cell context, including issues of scalability, interpretability, and integration with other analytical pipelines. We also explore emerging trends and opportunities, such as the use of TDA in multimodal and longitudinal single-cell studies, and the potential for incorporating topological priors into deep learning models.

Ultimately, this review seeks to bridge the gap between theory and practice by elucidating how TDA can enhance our understanding of single-cell data. As single-cell technologies continue to evolve and generate increasingly complex datasets, the ability to capture and interpret the topological features of these data will become increasingly essential. By highlighting the contributions and future potential of TDA, we aim to encourage its broader adoption and to inspire new avenues of research at the intersection of topology, computation, and biology. While several review articles have explored the application of topological data analysis (TDA) to biological systems in general [e.g., (6, 41, 42)], a comprehensive synthesis focused specifically on single-cell data modalities—including transcriptomics, proteomics, and spatial biology—is still lacking.

Our article addresses this gap by providing an integrative overview of TDA tools tailored to the unique challenges and opportunities presented by single-cell data: high dimensionality, sparsity, nonlinearity, and multimodality. We further emphasize biological interpretation, the use of TDA in realistic experimental contexts (e.g., cancer immunotherapy), and integration with established single-cell workflows. In doing so, we aim to offer both a conceptual and practical framework that complements prior general-purpose reviews, while providing actionable insights for researchers working directly with single-cell data.

In particular, unlike prior reviews, which often treat TDA as a generic tool across domains, our review examines how TDA methods are adapted, implemented, and interpreted in the context of specific biological use cases such as immune profiling, tissue architecture, and rare cell state identification.

## 2 Concepts and definitions

We will, first of all, introduce some essential concepts and mathematical notation that will be useful to develop understanding of the tenets, assumptions and applications of topological data analysis for the study of large complex data corpora (1, 2, 43) such as those prevailing in single cell biology.

### 2.1 Topological space

A *topological* sp*ace* is a set *X* along with a collection 𝒯 ⊆ 2*^X^* of subsets of *X*, called the *topology*, satisfying:

1. ∅ ∈ 𝒯 and *X* ∈ 𝒯,
2. The union of any collection of sets in 𝒯 is also in 𝒯,
3. The intersection of any finite number of sets in 𝒯 is also in 𝒯.

This structure defines notions of *continuity* and *nearness* without requiring a notion of distance. This will be extremely relevant in single cell biology analytics, for instance, in the context of cell clustering and cell type annotation.

### 2.2 Simplicial complex

A simplicial complex is a set composed of vertices, edges, triangles, and their higher-dimensional counterparts. Formally, a *finite abstract simplicial complex K* is a collection of subsets of a finite set *V* such that if *σ* ∈ *K* and *τ* ⊆ *σ*, then *τ* ∈ *K*. Elements *σ* ∈ *K* are called *simplices*.

**Figure 1:**
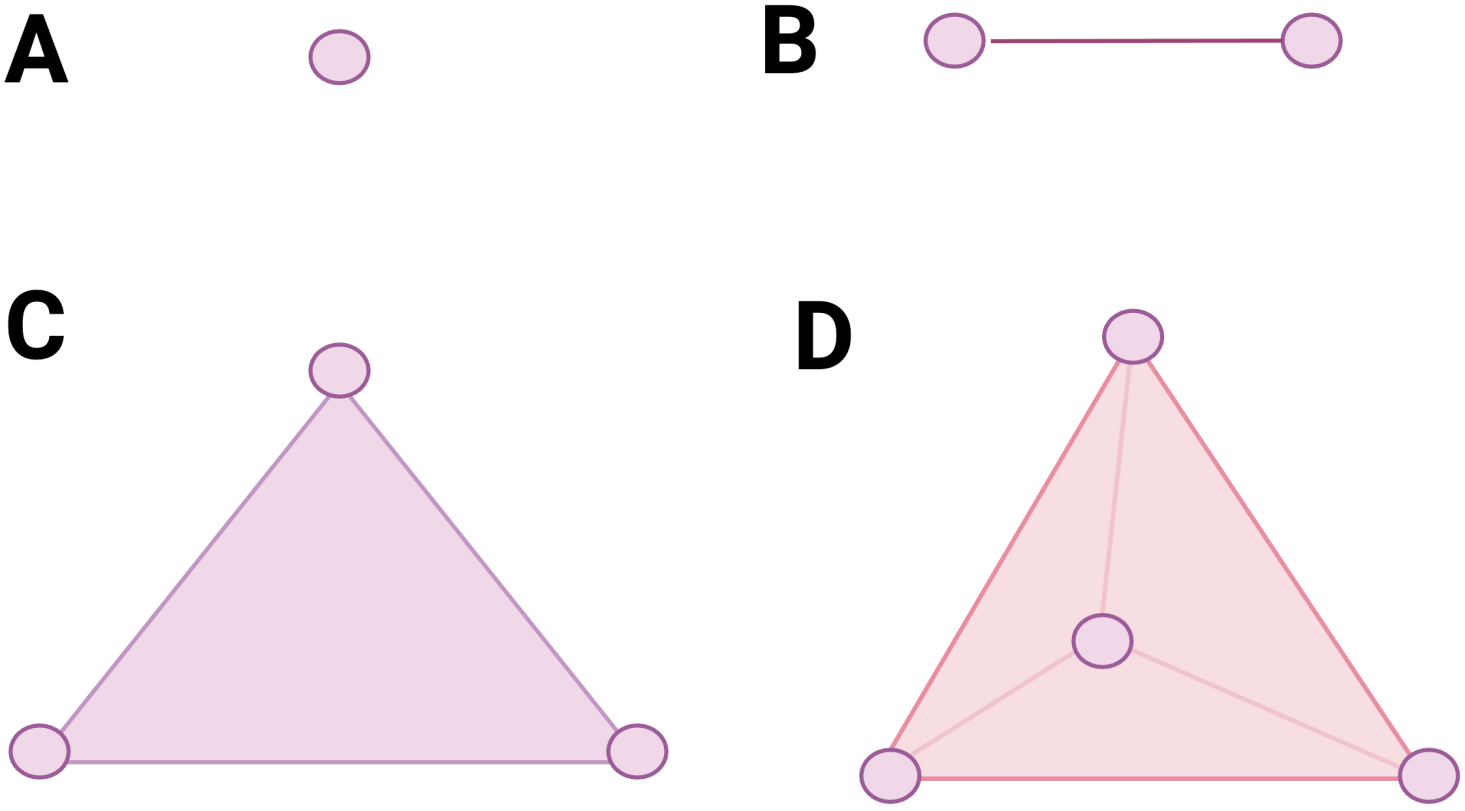
Some elementary simplices. **(A)** a 0-simplex, **(B)** a 1-simplex, **(C)** a 2-simplex, **(D)** a 3-simplex.

In **Figure 1** we can see some elementary simplices, namely:

- A 0-simplex is a point.
- A 1-simplex is an edge.
- A 2-simplex is a triangle.
- A 3-simplex is a tetrahedron.

### 2.3 Homology and Betti numbers

Homology is an algebraic method to detect *holes* in topological spaces in different dimensions.

The *k*-th homology group, *H_k_* (*X*), is an algebraic object (often a vector space over a field, such as ℤ2) that describes the *k*-dimensional holes in *X*. The Betti number *β_k_* is the rank of *H_k_* (*X*), i.e.,


$$\beta_{k}=\text{rank}\left(H_{k}\left(X\right)\right)$$

Interpretation:

- *β*_0_: is the number of connected components
- *β*_1_: is the number of 1-dimensional holes (loops)
- *β*_2_: is the number of 2-dimensional voids (cavities), etc.

**Figure 2:**
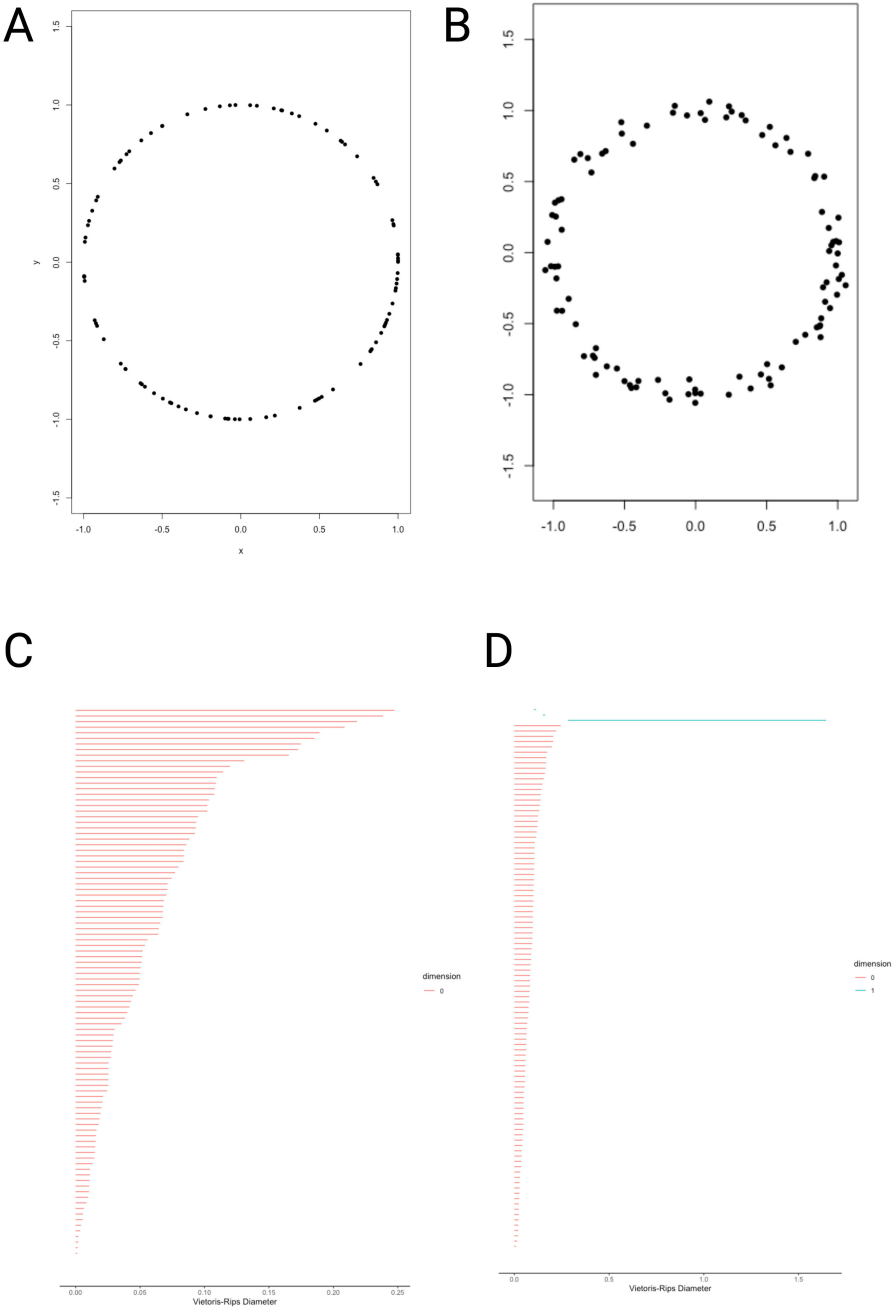
Using persistent homology to analyze two datasets. **(A)** presents a set of points sampled from a circle without noise, **(B)** presents another set of points sampled from a circle with some added noise, **(C, D)** present the homology barcode plots for each of these sets of points respectively. Notice the different scales of the x-axis in **(C, D)**.

In **Figure 2** we present a set of points sampled from a circle without noise (Panel A) and with some added noise (Panel B) forming two algebraic topological objects (spaces) and $X_{B}$ , respectively. By supplementing a simplicial complex structure we can analyze their homology.

. and can represent two different sets of measurements. By building the simplicial complexes and analyzing their related homology groups, we can notice some structure that may not evident just by looking at the sets of points.

### 2.4 Persistent homology

Persistent homology tracks the birth and death of topological features (like connected components, loops, and voids) across a *filtration*, which is a nested sequence of topological spaces:


$$\emptyset =X_{0}\subseteq X_{1}\subseteq \cdots \subseteq X_{n}=X$$

Each topological feature appears (is *born*) at some scale *ϵ_b_* and disappears (dies) at a later scale *ϵ_d_*. The persistence of a feature is *ϵ_d-_ϵ_b_*. These are often visualized in two different and equivalent ways:

- Persistence diagrams: Multisets of points $\left(\epsilon_{b},\epsilon_{d}\right)\in {\mathbb{R}}^{2}$

- Barcodes: Horizontal lines representing the lifespan of features

In **Figure 2C, D** we can see the barcode plots for the sets of points in panels A and B of the same figure. WE can notice that in **Figure 2C** (corresponding to points sampled from a noiseless circle), the only homology present is in dimension 0 (red bars) corresponding to the homology of connected components. **Figure 2D** presents a similar *β*_0_ homology (red bars), but also presents dimension 1 homology (blue bars, at the top of **Figure 2D**), corresponding to the presence of loops.

**Figure 3:**
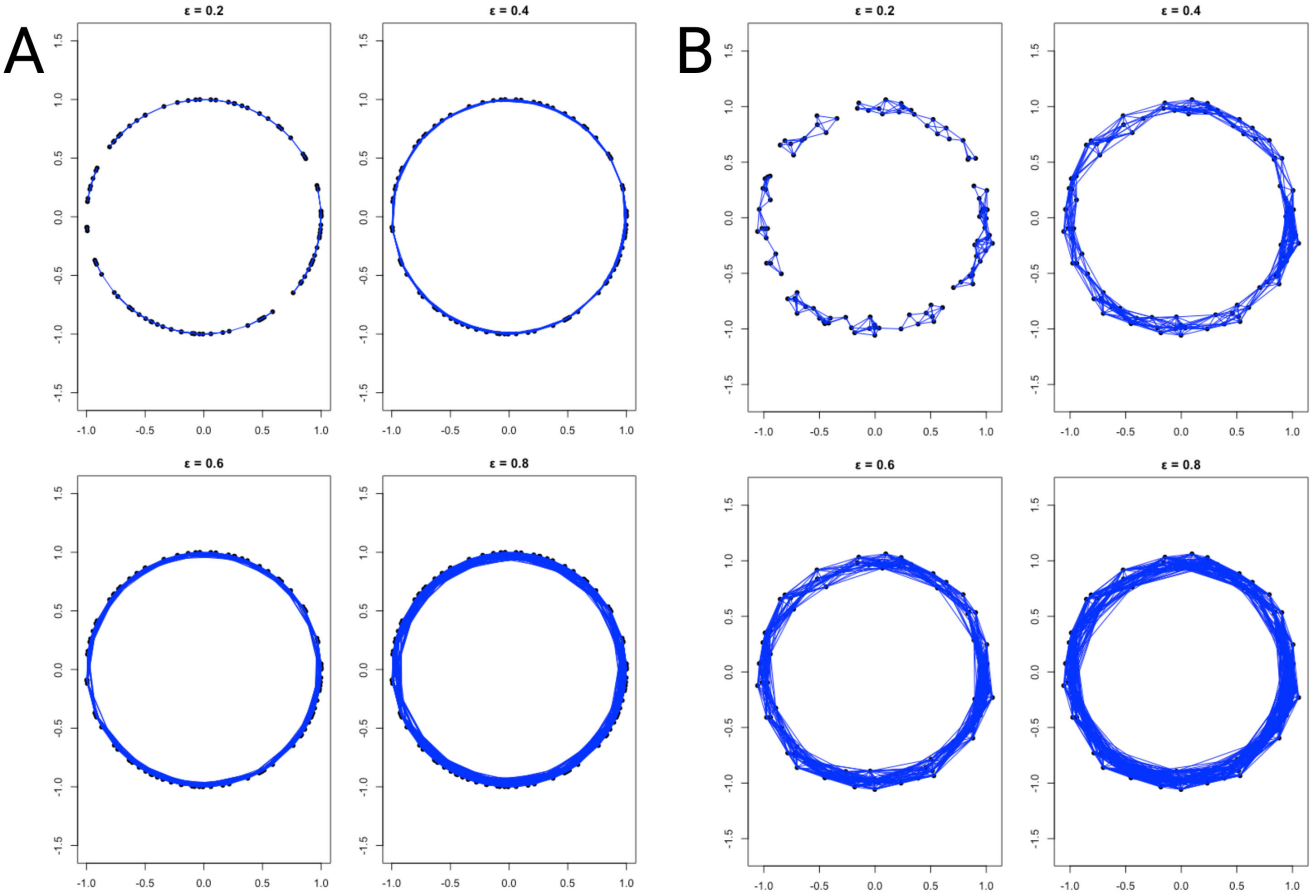
Persistence diagrams for the sets of points in **Figure 2A** and **Figure 2B**. **(A)** here presents the Persistence homology diagram for the 0-dimension homology of the set of points sampled from the noiseless circle (**Figure 2A**), whereas **(B)** shows the Persistence homology diagram for the 0- and 1- dimension homologies of the set of points sampled from the noisy circle (**Figure 2B**). In **(A)** only 0-dimensional homology is present (as in **Figure 2C**), whereas in **(B)**, 0- and 1- dimensional homology is shown (as in **Figure 2D**). (In both panels the diagonal is the identity line. Notice that due to different ranges in the X and Y dimensions the angle appears distorted. In reality, it is a 45*°* angle as expected from an identity line). Points closer to the diagonal are short lived (e.g. blue points here related to added noise), whereas points far from the identity line are *persistent*, likely related to distinctive features of the data.

**Figures 3A, B** presents the persistence homology diagrams for the data sets in **Figures 2A, B**, respectively. Persistence homology diagrams convey similar information as barcode plots, however, some features are more evident in one visualization or the other. Noise or short-lived features, for instance, are easier to see in persistence diagrams.

### 2.5 Vietoris–Rips complex

Given a set of points *P* ⊂ ℝ^*n*^ and a distance threshold *ϵ >* 0, the Vietoris–Rips complex VR*_ϵ_* (*P*) is a simplicial complex where a *k*-simplex is included if all pairwise distances among its *k* + 1 vertices are less than or equal to *ϵ*:


$${\text{VR}}_{\epsilon }\left(P\right)=\left\{\sigma \subseteq P❘∥x_{i}-x_{j}∥\leq \epsilon \;\mathrm{for}\;\mathrm{all}\;x_{i},x_{j}\in \sigma \right\}$$

This complex is widely used to build filtrations in persistent homology.

Intuitively, a Vietoris–Rips complex is a way to turn a set of data points into a geometric shape that reveals its underlying topological structure. Given a distance threshold (*ϵ* above), we connect points whose pairwise distances are within *ϵ*, forming edges. When sets of three points are all connected pairwise, we fill in the triangle between them; for four fully connected points, we add a tetrahedron, and so on. This process builds a simplicial complex (see above definition) that represents how the data is connected at that scale.

By varying *ϵ*, we get a sequence of these complexes – which is a filtration –that captures how topological features like clusters (*β*_0_), loops (*β*_1_), and voids (*β*_2_) appear and disappear as the scale changes. Persistent homology uses this filtration to identify which features persist across scales, helping distinguish true structure from noise. In single-cell data analysis, Vietoris–Rips complexes help detect clusters, developmental trajectories, or cycles in high-dimensional gene expression space, making them a powerful tool to understand complex biological relationships.

**Figure 4:**
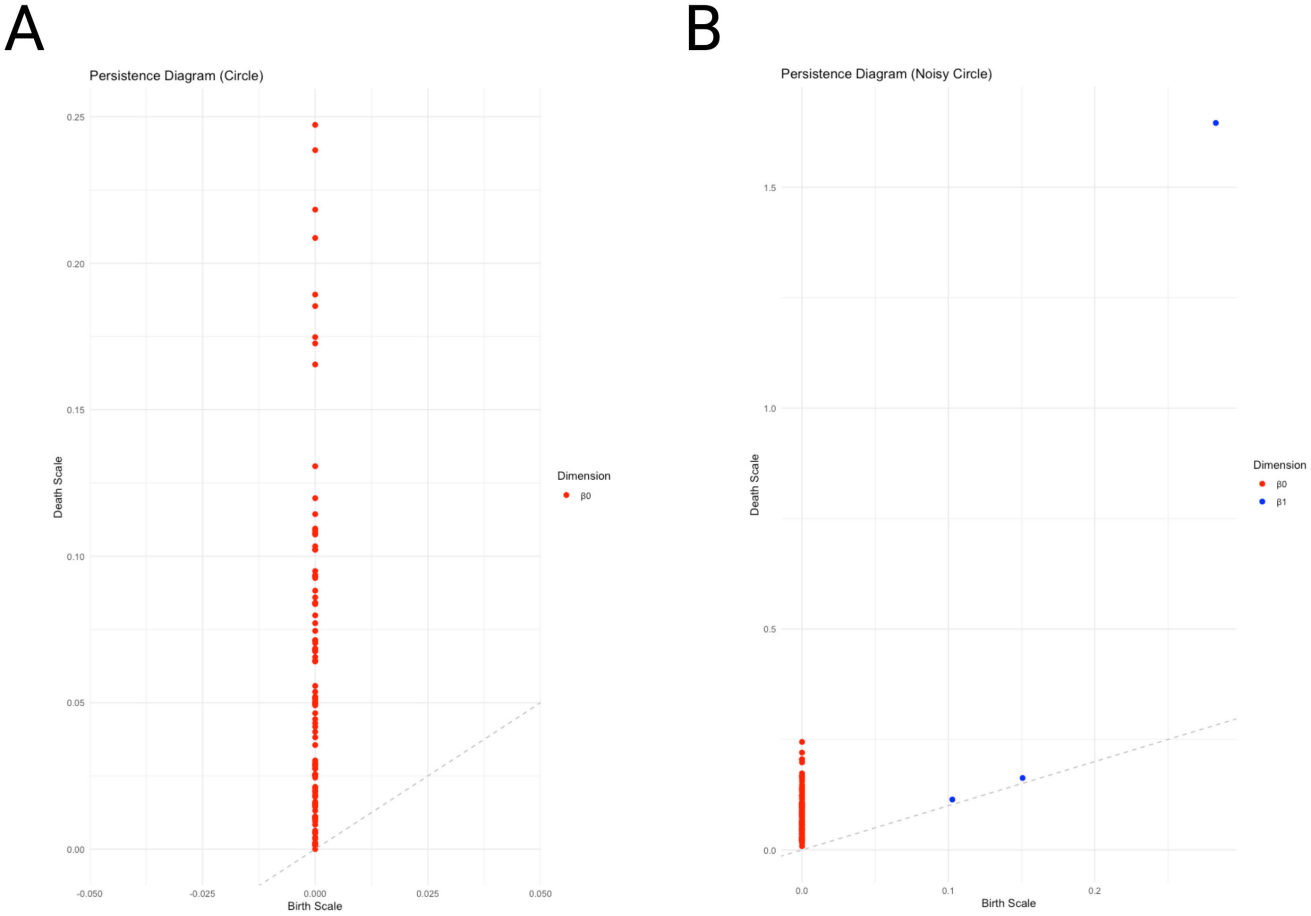
Vietoris Rips complexes corresponding to four different *ϵ* values (0.2, 0.4, 0.6 and 0.8) from the same datasets as in **Figure 2**. **(A)** presents the Vietoris Rips complex for the noise-less circle (**Figure 2A**), whereas **(B)** shows the Vietoris Rips complex for the noisy circle (**Figure 2B**).

**Figure 4** presents the Vietoris Rips complexes for the datasets in **Figures 2A, B** for four different values of *ϵ*. Although subtle noise was added, it was enough to change the homology features. It can be seen that higher order simplices appear in **Figure 4B**, as it can also been observed by the presence of *β*_1_ homology in **Figures 2D**, **3B**.

### 2.6 Mapper algorithm

The Mapper algorithm is a method for summarizing high-dimensional data by constructing a graph (or simplicial complex) that reflects its topological structure. Steps include:

1. Apply a filter function *f*: *X* → ℝ to the data.
2. Cover the range of *f* with overlapping intervals.
3. Cluster data points in the preimages of these intervals.
4. Build a graph whose nodes represent clusters and whose edges represent shared data points.

Mapper outputs a compressed topological representation of the data, capturing both local and global structure. The iterative mapping used in Mapper, where data is partitioned along a filter function and clustered locally, bears a superficial resemblance to manifold learning methods such as UMAP or t-SNE, in that both aim to reveal low-dimensional structure in high-dimensional data. However, the two approaches differ fundamentally in both goals and methodology.

**Figure 5:**
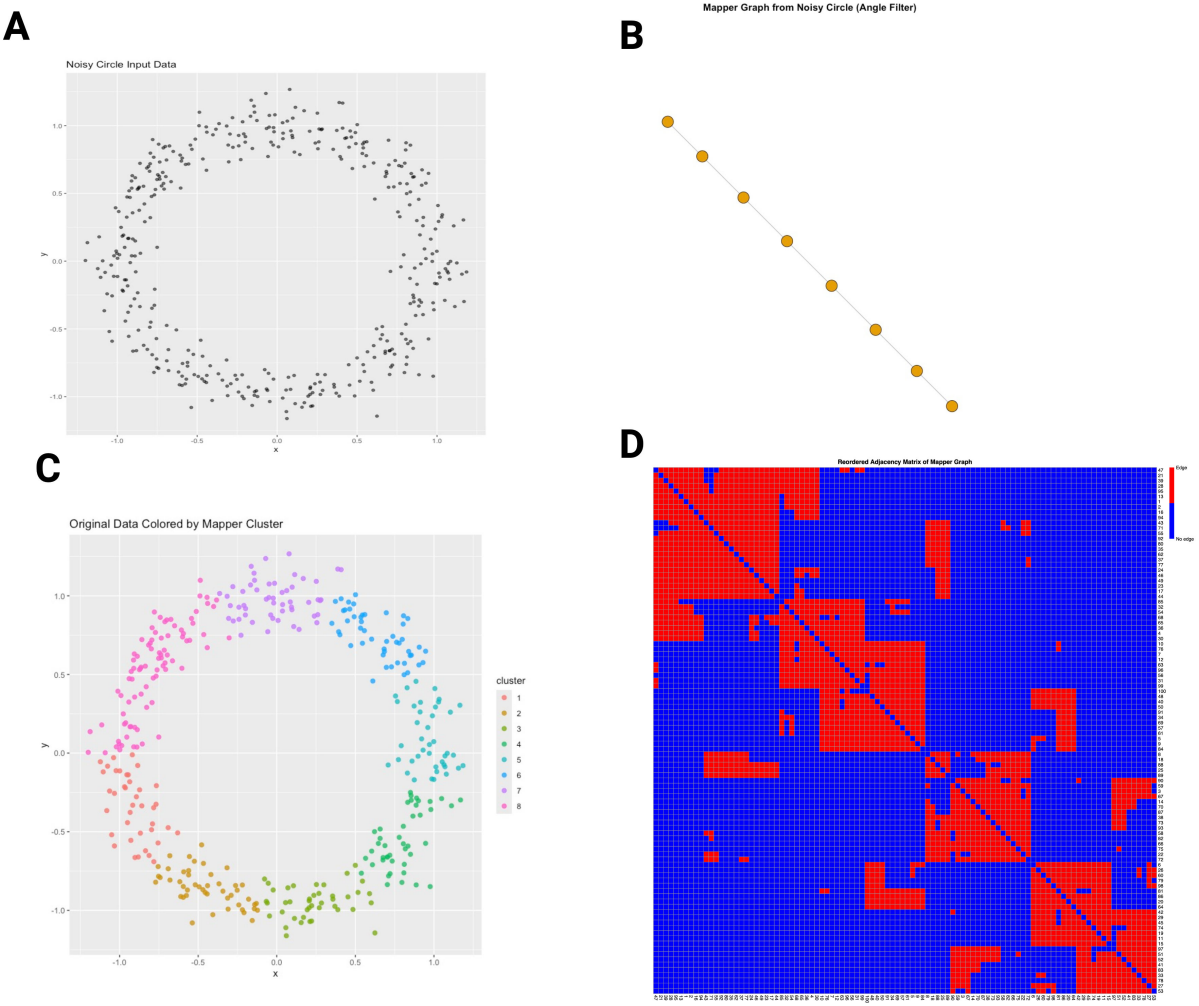
Illustration of the Mapper algorithm. **(A)** Original noisy circle point cloud. **(B)** Filter function assigns scalar values (e.g., angle). **(C)** Points in the datacloud clored according with the clusters generated by Mapper **(D)** Heatmap of the Mapper adjacency matrix encodes connectivity between clusters. Red cells indicate connections (edges) between clusters, while blue cells indicate no connection.

**Figure 5** illustrates the core steps of the Mapper algorithm. Panel A shows an example point cloud shaped like a noisy circle. Panel B demonstrates applying a filter function (e.g., angle or a principal component) that assigns scalar values to each point, effectively ordering the data. Panel C depicts dividing the filter range into overlapping intervals, within which local clustering identifies coherent groups of points. Finally, Panel D shows the resulting Mapper graph, where nodes represent clusters and edges indicate shared points between overlapping intervals, capturing the global topological structure of the data as a connected loop.

#### 2.6.1 Mapper versus UMAP

UMAP (Uniform Manifold Approximation and Projection), for instance, learns a single global low-dimensional embedding of the data by optimizing preservation of local neighbor relations across the entire dataset. It excels at visualization, providing a 2D or 3D layout that is highly interpretable for exploratory analysis. However, UMAP embeddings can distort global topology (for example, breaking loops or merging disconnected clusters), and they do not offer a formal topological summary.

By contrast, Mapper does not produce a single embedding, but constructs a graph reflecting the shape of the data across overlapping intervals of a chosen filter function. The resulting Mapper graph explicitly encodes connectivity and potential loops (i.e., 1-dimensional topological features), offering a compressed but topologically-informed summary of the data structure.

This difference is particularly valuable in single-cell analysis, where important biological variation can be cyclic or branched (e.g., cell cycle trajectories, lineage differentiation paths). Mapper and other TDA approaches can capture these higher-order structures more explicitly than UMAP, supporting hypothesis generation about underlying biological processes.

Thus, while UMAP remains the standard for quick, intuitive visualization, TDA-based methods provide complementary insights that formalize and preserve topological features in a way that projection-based embeddings may obscure.

### 2.7 Stability theorem (persistent homology)

The stability theorem for persistent homology ensures that small changes in the input data lead to small changes in the persistence diagram, measured using the *bottleneck distance*:


$$d_{B}\left(D_{1},D_{2}\right)=\underset{\gamma }{\inf}\underset{x\in D_{1}}{\sup}∥x-\gamma \left(x\right){∥}_{\infty }$$

where *D*_1_ and *D*_2_ are persistence diagrams, and *γ* ranges over all bijections between points in the diagrams (with points possibly matched to the diagonal).

This property makes persistent homology robust to noise.

Stability Theorem ((44)): Let f, g: *X*→ℝ be *tame* functions defined on a triangulable space X, and let D(f) and D(g) be their respective persistence diagrams.

Then: dB(D(f),D(g))≤∥f−g∥∞

where *d_B_* is the bottleneck distance and ‖ · ‖_∞_. is the supremum norm. This result ensures that small perturbations in the input data (or filtering function) lead to small changes in the persistence diagram, providing robustness of the topological summary to noise. This property is particularly relevant for single-cell data, where technical and biological noise is prevalent. The theorem provides theoretical support for the use of persistent homology in noisy biological contexts.

## 3 TDA in single cell transcriptomics

ngle-cell transcriptomic analysis, i.e. the study of gene expression patterns at the single-cells level, is arguably the most established and widely used approach in single-cell biology, despite inherent challenges such as sparsity, dropouts, and technical noise. Gaining biological understanding from the enormous quantity of high dimensional data provided by today’s single cell RNASeq experiments is a daunting task. Among the many available approaches to this problem, we believe TDA offers some advantages, especially in terms of interpretability and/or explainability. Below we will present an outline of how can we do so, in a quite general single cell gene expression analysis scenario (10–12).

Single-cell RNA sequencing allows the quantification of gene expression at the resolution of individual cells, producing high-dimensional datasets where each cell is represented as a point in a space defined by the expression levels of thousands of genes. These data are inherently sparse, noisy, and nonlinear due to technical artifacts, dropout events, and biological variability (21). TDA offers a unique set of tools to navigate these complexities (45, 46) and to reveal meaningful biological structure that may not be identified by conventional methods.

The first step in applying TDA to scRNA-seq data typically involves dimensionality reduction and normalization. Raw count matrices are transformed through log-normalization or more sophisticated variance-stabilizing transformations, and a subset of highly variable genes is selected to reduce noise (21). The resulting expression profiles, often embedded in a lower-dimensional space (e.g., via PCA or diffusion maps), serve as input for TDA. This transformation aims to preserve local and global structures relevant for inferring topological features from the data cloud; however, the quality of this preservation depends critically on the choice of filter function, cover parameters, clustering resolution, and the intrinsic geometry of the data.

Note that while Mapper is designed to capture meaningful topological features, the resulting graph can be sensitive to filter function choice, interval overlap, clustering resolution, and sampling density. Careful parameter tuning and validation are recommended for robust inference.

Persistent homology (47) is one of the most powerful tools within TDA for scRNA-seq analysis. By constructing a filtration (e.g., a Vietoris–Rips complex) over the cellular point cloud, we can identify topological features such as connected components (*β*_0_), loops (*β*_1_), and higher-dimensional voids (*β*_2_) (48). In a biological context, *β*_0_ corresponds to discrete subpopulations of cells, while *β*_1_ may capture circular or cyclic gene expression programs—such as those seen in cell cycle dynamics or oscillatory regulatory networks. Persistent homology thus provides a way to infer the global architecture of transcriptional landscapes in a scale-robust manner.

The Mapper algorithm, offers another approach for exploring the topological structure of scRNAseq data (26, 31–33). By applying a filter function—such as pseudotime scores, diffusion components, or pathway activation indices—Mapper projects the data onto a lower-dimensional axis and builds a network summarizing the connectivity of local clusters (24, 49). The resulting Mapper graph can reveal branching patterns indicative of developmental trajectories, bifurcations, and intermediate cell states. Unlike trajectory inference methods that impose linear or tree-like assumptions, Mapper flexibly captures multiple paths, cycles, and convergence points in the data.

These TDA tools have been used to uncover novel biological insights in various scRNA-seq studies. For instance, persistent homology has revealed multiscale structure in hematopoietic stem cell differentiation and enabled the identification of rare progenitor populations (50). Mapper-based analyses have traced alternative routes of epithelial cell maturation and characterized the plasticity of immune responses during infection or cancer (51, 52). Importantly, such analyses often reveal subtle relationships that evade detection by clusteringbased approaches, highlighting the continuity of cell state transitions and the topological complexity of gene expression spaces.

A key advantage of TDA in this context is its robustness to noise and parameter choice (2). Unlike clustering or manifold learning techniques that can be sensitive to tuning parameters and initialization, persistent homology offers a multiscale summary that is likely stable under small perturbations of the data (3). This makes it particularly well suited for single-cell transcriptomics, where dropout effects and measurement variability can significantly affect downstream interpretations.

As TDA methods mature, their integration with single-cell workflows is becoming increasingly streamlined. Hybrid approaches that combine TDA with graph neural networks, clustering, and differential expression testing are beginning to bridge the gap between topology and statistical inference (53). These developments are poised to enhance the interpretability and biological relevance of TDA outputs in transcriptomic studies.

Popular single-cell analysis suites like *Seurat* (R) and *Scanpy* (Python) do not include native tools for topological data analysis (TDA), but they can be integrated with external TDA libraries. Seurat itself does not include built-in TDA functionality. However, you can interface it with TDA tools in R such as:

- TDAmapper (https://github.com/paultpearson/TDAmapper): Implements the Mapper algorithm (54).
- TDA: Provides methods for computing persistent homology (e.g. Vietoris–Rips complexes, persistence diagrams, https://cran.r-project.org/web/packages/TDA/index.html).
- TDAstats (https://cran.r-project.org/web/packages/TDAstats/index.html): Simplifies computation of persistent homology using the GUDHI backend.

Data from Seurat objects (especially PCA-reduced data or UMAP embeddings) can be extracted and passed to these packages for TDA analysis.

Scanpy also does not implement TDA directly, but is compatible with Python-based TDA libraries such as:

- GUDHI (https://gudhi.inria.fr/): A powerful library for computing simplicial complexes, persistent homology, and persistence diagrams. It is commonly used for Rips complex construction and TDA pipelines (55).
- giotto-tda (https://github.com/giotto-ai/giotto-tda): A modern, scalable TDA library that integrates well with scikit-learn pipelines. It includes Mapper, persistence homology computation, and visualization tools (56).
- scTDA (https://github.com/CamaraLab/scTDA): Specifically designed for single-cell data, it integrates Mapper and uses diffusion maps for data representation before TDA. While not actively maintained, it offers a solid proof-of-concept (23).
- KeplerMapper (57) (see also https://github.com/MLWave/kepler-mapper): A user-friendly Python implementation of the Mapper algorithm, easily integrated with Scanpy-derived embeddings.

TDA thus offers a principled and flexible framework to explore the global structure of single-cell transcriptomic landscapes. By moving beyond linear assumptions and embracing the shape of the data, it enables a deeper understanding of how cell identities emerge, differentiate, and interact within complex biological systems. As new high-resolution and multimodal technologies further increase the richness of single-cell datasets, topological approaches are likely to play a central role in their analysis and interpretation.

It is important for users to understand the forms of output these TDA tools produce and how to interpret them. Packages implementing persistent homology (such as TDA, Gudhi, and Ripser) typically output persistence diagrams or barcodes. As we previously mentioned, these are 2D plots (or matrices of birth–death pairs) that summarize the lifetimes of topological features (e.g., connected components, loops, voids) across scales. Features that persist over large scale ranges (far from the diagonal) are typically interpreted as meaningful topological signals.

Tools for Mapper analysis (e.g., TDAmapper in R, KeplerMapper in Python) output graphs in which nodes represent clusters of data points (found in overlapping filter intervals) and edges connect nodes sharing samples. These Mapper graphs capture global data shape, including connectivity, branching, and cycles. The graph structure can be exported as an adjacency matrix or visualized interactively.

Some packages also provide embeddings or cluster assignments as outputs. For example, Mapperbased methods can be used as dimensionality reduction tools by laying out the Mapper graph in 2D for visualization. Users should interpret these outputs not simply as a low-dimensional projection, but as a topology-preserving summary of the data’s structure that can reveal branching trajectories, cycles, and other non-linear relationships not apparent in standard dimensionality reductions like PCA or UMAP.

Overall, careful interpretation of these outputs – in combination with domain knowledge – is crucial to extracting meaningful biological insights.

### 3.1 Advantages and limitations of TDA in single cell transcriptomic analysis

Applying TDA to single-cell transcriptomics offers a transformative approach to uncovering, for instance, rare cell populations, transitional states, and complex branching trajectories within highdimensional gene expression landscapes (58, 59). Traditional clustering and dimensionality reduction methods often assume discrete, well-separated clusters or linear transitions between cell types, which can obscure the subtle, continuous, and often nonlinear nature of cellular differentiation and identity. TDA, by contrast, considers the intrinsic shape of data, capturing both local features and global connectivity, thus providing a richer and more *faithful* representation of cellular heterogeneity (6).

One of the central strengths of TDA here lies in its ability to identify rare cell populations (50). These subpopulations may occupy small, isolated regions in the high-dimensional space of gene expression and are often overlooked by clustering algorithms that rely on density or global structure (51). Through persistent homology, TDA is able to detect small but topologically significant features—such as distinct connected components that persist across multiple scales of analysis—indicating the presence of biologically meaningful outlier groups. These rare populations might correspond to stem cells, transient progenitor states, or disease-associated phenotypes, and their detection is critical for understanding developmental biology, immune responses, or cancer heterogeneity.

TDA also excels in revealing transitional states that lie between well-defined cell identities. In developmental or dynamic processes, cells do not transition abruptly from one state to another, but rather traverse a continuum of intermediate configurations (52). Mapper graphs and persistence diagrams can capture these transitions by visualizing how cells are organized along continuous or looping paths, rather than forcing them into discrete categories. This allows researchers to pinpoint regions of *transcriptional plasticity* (60) where cells are in flux—actively differentiating, reprogramming, or responding to stimuli—offering insights into the mechanisms that govern cell fate decisions (34, 61).

Furthermore, the topological structure of scRNA-seq data often includes branching trajectories, where progenitor cells differentiate into multiple lineages through bifurcations or more complex branching events. Standard trajectory inference methods typically model such processes as trees or linear paths, but may struggle with cyclic, convergent, or multifurcating structures (62–64). TDA, particularly via the Mapper algorithm, provides a flexible way to represent these patterns without imposing restrictive assumptions. The resulting graphs naturally capture the geometry of branching and looping, reflecting the multiplicity of developmental routes and the possibility of reversion or convergence between cell states (65, 66).

Approach such as Mapper are not without disadvantages. The results of Mapper are highly sensitive to parameter choices, including the filter function, the number of intervals, and the overlap percentage (67). These parameters often require empirical tuning and can influence the shape and connectivity of the resulting graph, potentially introducing subjectivity. Furthermore, the biological interpretation of topological features, such as loops or branches, can be nontrivial and may require additional validation using experimental or orthogonal computational methods. Lastly, while Mapper is effective for visualization and hypothesis generation, it may not provide rigorous statistical assessments or p-values associated with observed features, necessitating downstream modeling or testing to substantiate findings (68).

It is also worth noting that other graph-based approaches for single-cell data analysis exist, such as PAGA (66), which is widely used for trajectory inference. Unlike Mapper, which uses a filter function and clustering to explicitly capture global topological features (including loops), PAGA models data as a k-nearest neighbor graph and abstracts it to a simplified connectivity graph capturing branching structures. Including such methods in the analytical toolbox helps provide a broader topological perspective on single-cell data.

By capturing the multiscale topology of gene expression data, TDA complements and enhances existing single-cell analysis frameworks. It enables us to discern structures that are biologically relevant but difficult to detect with *classical* tools. In doing so, TDA opens new avenues for understanding the complexity of cellular ecosystems, the emergence of functionally distinct phenotypes, and the plasticity inherent to many biological processes. Its capacity to handle noise, sparsity, and nonlinear geometry makes it particularly well-suited to the unique challenges posed by single-cell data, and its continued integration into biological workflows is likely to yield novel discoveries across developmental biology, immunology, and regenerative medicine (6).

Despite its many advantages, TDA also presents several limitations when applied to the study of single-cell transcriptomics. One of the primary challenges is the sensitivity of TDA methods to preprocessing choices, including normalization, dimensionality reduction, and gene selection (26). Since TDA operates on point cloud data derived from these upstream transformations, inconsistencies or biases introduced at this stage can propagate into the topological summaries. For example, the use of different distance metrics or embedding techniques can significantly alter the geometry of the data and, consequently, the resulting persistence diagrams or Mapper graphs. This dependency necessitates careful and often dataset-specific optimization, which can hinder the standardization and reproducibility of TDA-based workflows (2).

Another limitation is the interpretability of topological features. While persistent homology captures robust multiscale structures in the data, translating these features into biological meaning is not always straightforward (69). For instance, the presence of a longpersisting 1-dimensional hole (*β*_1_) may suggest a cyclic process such as the cell cycle, but assigning this feature to a specific biological pathway or regulatory mechanism requires additional analysis, such as overlaying metadata or incorporating prior knowledge. Moreover, the biological relevance of short-lived or higher-dimensional features (*β*_2_, *β*_3_, etc.) is still an open question in many contexts. As a result, researchers may need to integrate TDA with complementary statistical or machine learning tools to derive actionable insights (20, 65, 70, 71).

Finally, computational scalability and parameter selection remain active areas of development for TDA methods (72–74). Persistent homology and Mapper algorithms can become computationally expensive as the number of cells and genes increases, particularly in the presence of large, high-resolution datasets typical of modern single-cell studies. Choosing appropriate filtration functions, cover parameters, and clustering resolutions often requires manual tuning and domain expertise, and there is no universally accepted strategy for parameter selection. These constraints may limit the accessibility of TDA to non-expert users and present obstacles to its integration into high-throughput pipelines. Addressing these limitations through better visualization tools, automated parameter tuning, and scalable algorithms will be critical to ensuring that TDA can reach its full potential in single-cell transcriptomics.

In this section, we aim to highlight a representative set of TDA tools that are actively maintained, widely used in the community, or particularly well-suited to single-cell data analysis workflows. Our selection is not exhaustive, but emphasizes tools that are either general-purpose (e.g., TDA, Gudhi, Ripser) or specifically designed with biological data, and in some cases single-cell modalities, in mind. We recognize that the landscape is rapidly evolving, and new packages such as scGeom (36) continue to expand the options available for applying TDA in single-cell analysis.

**Table 1** describes some tools surveyed, as well as their typical output, and practical considerations for users. This aims to help readers select appropriate tools for their specific data and analysis goals, while also acknowledging the trade-offs and limitations inherent to each approach.

**Table 1:** Comparison of TDA tools, their outputs, advantages, and limitations.

Tool	Language	Type of TDA	Typical Output	Pros	Limitations
TDA	R	Persistent Homology	Persistence diagrams, barcodes	Easy integration with R workflows; classic PH analysis	Requires manual parameter tuning
Gudhi	Python	Persistent Homology, Mapper	Diagrams, barcodes, simplicial complexes	Very flexible, efficient, broad features	More coding required; steeper learning curve
Ripser	Python/R	Persistent Homology	Diagrams, barcodes	Extremely fast PH computation	Minimal built-in visualization
TDAmapper	R	Mapper	Graph (adjacency matrix)	Simple Mapper implementation; easy for small datasets	Less scalable; fewer advanced options
KeplerMapper	Python	Mapper	Graph (JSON, networkx)	Good visualization support; widely used	Parameter-sensitive; can be hard to tune
scGeom	Python	Single-cell tailored TDA	Mapper graphs, PH diagrams, embeddings	Designed for single-cell data; integrated with scanpy workflows	Newer; documentation still developing

## 4 TDA in single cell proteomics

The emergence of single-cell proteomics, particularly through techniques such as mass cytometry (CyTOF) (13, 14), CITE-seq (75–77), and imaging mass spectrometry (78, 79), has enabled the measurement of protein expression at single-cell resolution with increasing depth and throughput. Unlike transcriptomics, single-cell proteomics captures the functional output of gene expression, offering a closer view of the cellular phenotype and dynamic signaling events (80, 81). This modality poses unique analytical challenges, including technical variability, lower feature dimensionality compared to transcriptomics, and complex inter-marker dependencies. TDA provides a robust framework to address these challenges by uncovering the underlying geometric and topological structure of protein expression spaces across individual cells.

In single-cell proteomics, each cell can be represented as a vector in a space defined by a selected panel of protein markers, which may include surface receptors, intracellular signaling molecules, and functional state indicators. These markers often exhibit intricate co-expression patterns and hierarchical regulation, reflecting the combinatorial nature of cell signaling and phenotypic plasticity. TDA, through tools like Mapper and persistent homology, can capture these multi-dimensional relationships and reveal subtle distinctions between phenotypic states that are not well separated by linear projections or clustering. For example, Mapper has been successfully applied to CyTOF data to identify functional subsets of immune cells and to map branching trajectories in response to external stimuli, such as cytokine exposure or checkpoint blockade therapies (82, 83).

One of the particular strengths of TDA in single-cell proteomics lies in its ability to highlight dynamic and transitional processes (84). Protein-level data are inherently more reflective of temporal changes, such as post-translational modifications and activation states. Mapper can capture these transitions in the form of topological paths or loops in the data graph, which may correspond to signaling cascades, response gradients, or cellular adaptation processes. Moreover, by using specific markers as filter functions—such as phosphorylation levels or surface activation markers— (81, 85, 86) researchers can guide the topological representation toward biologically interpretable features, enhancing the explanatory power of the analysis. As the field of single-cell proteomics evolves to include more spatial and time-resolved measurements, TDA is poised to play an increasingly central role in decoding the shape of proteomic landscapes at the single-cell level.

## 5 TDA in spatial biology

Spatial biology technologies, including spatial transcriptomics (19, 71), multiplexed immunofluorescence (87–89), imaging mass cytometry (15, 82), and MERFISH (90–92), enable the simultaneous measurement of molecular profiles and spatial coordinates across tissues at single-cell or even subcellular resolution. These data offer unprecedented opportunities to study how cells are organized in space, how microenvironments influence cell states, and how structural features of tissues relate to physiological or pathological processes. However, the high dimensionality, spatial heterogeneity, and complexity of these datasets also introduce significant analytical challenges. TDA with its ability to characterize the shape and connectivity of data across scales, offers a promising approach to uncovering spatial patterns and relationships that may be difficult to capture with conventional spatial statistics or clustering techniques (37, 93, 94).

A key advantage of TDA in spatial biology is its ability to integrate both molecular and spatial information into a unified topological representation. By incorporating cell positions into the construction of simplicial complexes – e.g. through *α*-complexes or witness complexes – (95) TDA may, for instance, reveal how gene or protein expression patterns are distributed and interact within the tissue microarchitecture. Persistent homology, in this context, could be be used to detect spatial domains, voids, gradients, or boundary structures, which may correspond to functionally distinct regions, barriers between tissue compartments, or signaling niches. These features may then be quantified across multiple spatial scales, potentially enabling researchers to explore the hierarchical organization of tissue without the need for arbitrary thresholds or rigid domain definitions (96–98). Of course, other non-biologically relevant features of the date may influence the properties of witness complexes, so caution needs to be taken during these analyses.

Moreover, TDA is particularly well-suited to uncovering spatial transitions and topological signatures associated with disease. In tumor microenvironments, for example, persistent homology can detect disruptions in tissue organization, emergent immune cell infiltration patterns, or the breakdown of structural compartmentalization (98–100). Mapper-based representations can further capture how spatial neighborhoods of cells relate to one another in terms of molecular similarity, forming graphs that reflect the flow of biological information or gradients of cellular activation across space (25, 101). This topological perspective is especially valuable in contexts where cellular behavior is not determined solely by intrinsic molecular states, but also by local context, spatial proximity, and interaction with surrounding cells and extracellular matrix.

As spatial biology continues to expand with higher resolution, multimodal platforms, and largescale atlases, the integration of TDA offers a scalable and conceptually rich approach to analyze spatially-resolved single-cell data. Its capacity to identify and quantify structural complexity—both within and across tissue compartments—positions it as a powerful complement to emerging computational frameworks in spatial systems biology.

## 6 TDA paves the way to uncover new biology

As we have discussed, TDA offers a unique and powerful lens through which to uncover previously hidden biological complexity. Unlike traditional methods that often rely on linear assumptions or discrete clustering, TDA enables the discovery of alternative cellular differentiation paths that may exist alongside canonical trajectories (102–104). In developmental biology, for example, cells do not always follow a single predetermined path to a mature state; instead, they may diverge, converge, or follow looping paths influenced by microenvironmental cues or stochastic gene expression (23, 39). TDA, particularly through Mapper and persistent homology, can capture these complex structures by preserving non-linear and higher-order relationships in the data. This makes it possible to identify parallel differentiation routes, detours, or even reversible transitions that would otherwise be missed, offering a richer understanding of cellular plasticity and fate decisions.

Beyond differentiation, TDA provides a framework to reveal the intricate architecture of tissues as a multi-scale, interconnected system. Spatial biology and single-cell technologies together generate high-dimensional spatially-resolved datasets, which encode not only the molecular state of each cell but also its physical context within a tissue. TDA can parse this dual information to map gradients, boundaries, and organizational motifs across tissues (105, 106). Persistent homology, for example, can detect voids, folds, or nested compartments that reflect the physical and functional structuring of tissue. These topological features often correlate with physiological functions or pathological states—such as the compartmentalization of immune responses in inflamed tissues, or the disruption of epithelial barriers in tumors—thus providing biologically meaningful abstractions of tissue complexity (107–109).

In the era of multimodal profiling, where transcriptomics, proteomics, epigenomics, and spatial data are integrated at the single-cell level, TDA offers a principled way to build interpretable models that reconcile these heterogeneous data types (6, 103). By representing data in topological structures such as simplicial complexes or Mapper graphs, TDA can serve as a common coordinate system onto which different modalities are projected. This allows researchers to identify correspondences across data layers, not simply at the level of cell types or clusters, but at the level of shared structural features, such as conserved trajectories or overlapping spatial patterns. The topological approach is especially well-suited for interpreting subtle or high-dimensional multimodal signals that evade intuitive visualization or single-modality analysis (72, 110). Beyond transcriptomic spot-level data, TDA approaches also have the potential to jointly analyze topological features of nuclear morphology and intercellular spatial relationships together with single-cell gene expression profiles (111). Such integrative analyses are particularly relevant as spatial omics advances to include multiplexed imaging and digital pathology resources, enabling a richer characterization of tissue architecture and cellular phenotypes.

Importantly, TDA’s capacity to globally preserve the structure of the data, while being robust to noise and data sparsity positions it as an ideal exploratory tool for hypothesis generation. Novel topological features, such as long-lived loops or persistent cavities, often prompt fresh biological questions: What does this loop represent in terms of cellular behavior? Is this cavity indicative of a physical tissue boundary, or an absence of a particular cell type? TDA can thus drive experimental inquiry by revealing features that are unexpected, difficult to define *a priori*, or missed by standard statistical summaries. In this way, topological analyses do not merely interpret known biological frameworks, but actively expand them (6).

Ultimately, the value of TDA lies in its philosophical shift: it approaches biological data not just as collections of points to be labeled or classified, but as shapes to be studied. In some sense, TDA *contextualizes* data points with respect to other points. This shift has profound implications. It allows researchers to uncover subtle organization in messy, high-dimensional data, to connect disparate biological signals across scales and modalities, and to construct models that respect the inherent geometry of living systems. As biological datasets continue to grow in complexity, and as the field moves toward more integrative and mechanistic understandings, TDA stands out as a method not only for analysis, but for discovery (41, 42, 112).

## 7 A TDA approach to systems immunology

One area of contemporary biology which will be extremely benefited by the combination of topological data analysis and single cell experimental approaches is systems immunology.

Imagine we are interested in the study of the immune response to checkpoint blockade immunotherapy in cancer (113). In this context, we aim to understand how individual immune cells, especially T cells, respond to treatment, differentiate over time, and adopt functional phenotypes associated with therapeutic success or failure (34, 114). A particularly notable application we can envision is the use of the Mapper algorithm to analyze scRNA-seq profiles of tumor-infiltrating lymphocytes (TILs) (115), enabling the identification of rare subpopulations of T cells with distinct transcriptional programs that correlate with treatment response.

In this approach, each T cell’s transcriptomic profile would represented as a point in highdimensional gene expression space (113). Mapper can be used to capture the shape of the data manifold potentially reflecting cellular trajectories, bifurcations, and loops corresponding to different immune cell states or differentiation programs.

This last (hypothetical) example is indeed not far from what already has been done. When applied to TILs, Mapper has revealed alternative activation states of CD8^+^ T cells, including exhausted phenotypes, memory-like precursors, and transitional intermediates that were previously obscured using standard clustering or linear dimensionality reduction methods (116).

The advantages of using TDA in this systems immunology context are several. First, TDA can – as we discussed previously – reveal continuous and branching trajectories of T cell differentiation and activation, offering a more nuanced view of immune heterogeneity than rigid clustering approaches (117). This is particularly valuable in immunology, where cell states often exist along a spectrum rather than in discrete categories. Second, TDA is robust to noise and dropout (46), common challenges in scRNA-seq, due to its focus on persistent features that remain across multiple scales of resolution. Third, Mapper outputs are visually interpretable and can integrate metadata –such as treatment response, cytokine production, or receptor expression – allowing researchers to spatially localize and annotate subpopulations within the topological graph (104, 114). This integrative capability aligns well with the goals of systems immunology, which seeks to understand the global coordination of immune responses.

The use of TDA in scRNA-seq data for systems immunology however, offers a compelling method to uncover hidden structures in complex immune responses, especially in dynamic settings such as cancer immunotherapy. By preserving the shape of immune cell trajectories and capturing transitional states, TDA enhances our ability to decipher the regulatory logic of immunity and to identify novel targets or biomarkers of treatment efficacy. Despite the methodological challenges, the interpretability and discovery potential of TDA make it a valuable addition to the computational immunologist’s toolkit.

## 8 Conclusions and perspectives

Topological Data Analysis is rapidly emerging as a powerful framework for the exploration of complex biological data, offering insights that extend beyond the capabilities of traditional linear and cluster-based methods. As single-cell technologies continue to evolve toward higher dimensionality, spatial resolution, and multimodal integration, the need for methods that can faithfully capture the intrinsic structure of these datasets becomes increasingly critical. TDA, grounded in mathematical topology, provides precisely such a framework—capable of preserving global data geometry, identifying subtle transitions, and quantifying relationships that are often missed by conventional approaches.

One exciting avenue for TDA in single-cell biology research is its integration with advanced machine learning frameworks. Graph neural networks (GNNs) are naturally suited to process the graph outputs of Mapper or other TDA constructions, potentially enabling more powerful downstream prediction or classification. Reinforcement learning and adversarial models can help optimize filter functions or clustering strategies to reveal biologically relevant topological features. Large language models (LLMs), with their capacity to encode complex multimodal knowledge, may eventually assist in annotating and interpreting topological summaries in a biologically informed manner. Integrating TDA with these multilayer models offers a path toward more interpretable, automated, and robust single-cell analysis pipelines, bridging the gap between mathematical topology and practical biological insight.

Looking ahead, the integration of TDA with machine learning, probabilistic modeling, and causal inference promises to deepen its utility in biological research. These hybrid models could enhance the interpretability of complex systems by embedding topological summaries into predictive frameworks, facilitating the construction of biologically grounded models that are both data-driven and theoretically robust. Additionally, the continued development of scalable TDA algorithms, better parameter selection heuristics, and more intuitive visualizations will be essential to broaden accessibility and adoption within the life sciences community.

Despite challenges in embedding TDA within end-to-end single-cell analysis pipelines, recent methods have begun to address this gap. For example, scGeom (36) and Gene2role (118) both apply TDA concepts – specifically, cluster embeddings and topological summaries – to reveal unique structural characteristics of gene regulatory network (GRN) modules reconstructed from single-cell omics data. Such approaches highlight the growing potential for TDA to provide interpretable, biologically relevant features in complex multi-omics analyses.

Embedding TDA into end-to-end single-cell analysis pipelines has also resulted hard to implement, recent deep learning methods have begun to address this gap. For example, scMGCA (119) uses graph convolutional networks to integrate gene expression and cell-cell PPMI matrices, extracting major gene signals and cellular topology into latent representations for downstream decoding. Similarly, methods such as scPrisma (120) and scGAE (121) use graph and manifold structures to learn meaningful low-dimensional embeddings. These approaches highlight the promise of combining topological insights with modern deep learning architectures to improve interpretability and predictive power in single-cell analysis.

Beyond single-cell transcriptomics, proteomics, and spatial omics, single-cell epigenomic modalities present additional opportunities for TDA. Techniques such as ATAC-seq, single-cell Hi-C, and RNA secondary structure sequencing such as KARR-seq (122, 123) generate high-dimensional data with inherently topological and regulatory interactions. For example, TDA frameworks could be used to define or refine spatial DNA topological associating domains (TADs) and elucidate their regulatory interactions (124). Incorporating TDA into single-cell epigenomics could thus provide new insights into the 3D genome organization and regulatory landscapes at single-cell resolution.

An important practical consideration is the computational scalability of TDA methods with increasing single-cell or spatial resolution. For example, the construction of Vietoris–Rips complexes for persistent homology typically has combinatorial scaling with the number of data points, making naive approaches infeasible for large datasets. Similarly, Mapper workflows involve repeated distance computations and clustering steps that can scale quadratically or worse with data size. This non-linear growth in computational cost underscores the need for efficient approximations, sparse filtrations, and scalable implementations, especially as single-cell and spatial transcriptomics datasets continue to grow in size and resolution.

As TDA matures, its potential to generate biologically meaningful hypotheses across disciplines—from developmental biology to immuno-oncology and regenerative medicine—is only beginning to be realized. By reframing how we conceptualize cellular organization and tissue complexity, TDA invites a new language for interpreting biology: one that embraces continuity, shape, and structure as foundational elements of understanding living systems.

## Appendix: code examples to perform selected TDA analytics in single cell data

Below are the links of some code repositories with examples of how topological data analysis has been applied in the context of single cell biology in diverse settings:

1. Github repository to reproduce the figures in this review (except by **Figure 1** that was manually drawn) https://github.com/CSB-IG/TDA_Single_Cell. These are just illustrative examples with no original, published or unpublished data.
2. Github repository to reproduce the PH analysis of (109): https://github.com/DrMSAbdullahi/PBMC_RNASeqHCC_PH_Analysis
3. Gitlab repository of the general analysis strategy https://gitlab.com/kfbenjamin/topact and Zenodo repository for the specific analyses https://doi.org/10.5281/zenodo.11050996 to reproduce the results of (96)
4. Github repository to reproduce the TDA of (115): https://github.com/bru08/ly_decount
5. Github repository of the code and data to reproduce the analysis of (113): https://github.com/salvadorchulian/shapecancerrelapse
6. Github repository of the code used in (101): https://github.com/kemplab/TDA-Microscopy-Pipeline
7. Github repository of the code used in (29): https://github.com/pqiu/Quantifying_clusterness_trajectoriness
8. Github repository of the code used in (121): https://github.com/ZixiangLuo1161/scGAE
9. Python and Julia code to reproduce the analyses on (100) can be found here https://github.com/irishryoon/multiplex_relations and here https://github.com/irishryoon/Dowker_persistence, respectively.
10. GitHub repository for code accompanying (98) can be found here https://github.com/MultiparameterTDAHistology/SpatialPatterningOfImmuneCells
11. Code repository to perform PAGA analytics (66) within the Scanpy software ecosystem (70) can be found here https://github.com/theislab/paga
12. Code repository, with tutorials and examples to perform scMGCCA https://pypi.org/project/scMGCA as used in (119) can be found here: https://github.com/Philyzh8/scMGCA
13. Github repository with the source code of sc-MTOP as performed in (111) is available here https://github.com/fuscc-deep-path/sc_MTOP and here https://doi.org/10.5281/zenodo.8364420
14. Code for the analyses performed in (69) is here https://github.com/jzthree/quasildr. An *Ocean* capsule tutorial can be found here https://codeocean.com/capsule/9866535/tree/v1
